# Observational Study of the Association between TCM Zheng and Types of Coronary Artery Stenosis: Protocol of a Multicenter Case Series Study

**DOI:** 10.1155/2018/2564914

**Published:** 2018-06-04

**Authors:** Chao Liu, Guang Chen, Dan Liu, Shaoping Nie, Xiao Wang, Xinsheng Huang, Haitao Li, Yi Li, Shihua Luo, Guoan Zhao, Fei Lin, Haoqiang He, Jun Li, Yanwei Xing, Zhenpeng Zhang, Jie Wang

**Affiliations:** ^1^Department of Cardiology, Guang'anmen Hospital, China Academy of Chinese Medical Sciences, Beijing 100053, China; ^2^Beijing University of Chinese Medicine, Beijing 100029, China; ^3^Beijing Anzhen Hospital, Capital Medical University, Beijing 100029, China; ^4^Yunnan Provincial Hospital of Traditional Chinese Medicine, Yunnan 650021, China; ^5^The First Affiliated Hospital of Xinxiang Medical University, Henan 453199, China

## Abstract

**Background:**

Coronary artery stenosis is the major pathological change of coronary heart disease (CHD). Within the framework of traditional Chinese medicine (TCM) theory, some kinds of TCM Zheng could exist in patients with CHD; accordingly, TCM practitioners could provide appropriate TCM therapy. However, little is known about the association between TCM Zheng and types of coronary artery stenosis. Such knowledge could help improve the accuracy and effectiveness of efforts to combine CHD treatment with TCM therapy. Therefore, the aim of this study is to determine the association between TCM Zheng and types of coronary artery stenosis.

**Methods and Design:**

This is a multicenter, large sample, case series study in 4 tertiary A hospitals from 3 provinces in China. A total of 3,000 eligible patients diagnosed with atherosclerosis or CHD and selected to undergo coronary angiography (CAG) will be enrolled in this study. We will use electronic case report forms (eCRF) to collect information, including baseline characteristics, TCM symptoms, CAG results, and GRACE scores according to standard operating procedures (SOP). Data will be analyzed by SPSS 20.0.

**Ethics:**

This study is approved by the Ethics Committee of Guang'anmen Hospital of the China Academy of Chinese Medical Sciences (no. 2017-058-KY-01) and is registered with chictr.org (registration number ChiCTR-ROC-17013221).

## 1. Introduction

Coronary heart disease (CHD) is currently the leading cause of death worldwide. According to an analysis of the causes of death and disease burden in the global population over the past 20 years, the number of deaths caused by CHD worldwide was 7,029,300 in 2010, accounting for 13% of total deaths [1]. According to the 2016 report on cardiovascular disease in China, the number of deaths from CHD was 11 million in 2015, the death rate of CHD in urban residents was 110.67/100 thousand, and the mortality rate of rural residents was 110.91/100 thousand in 2015. In addition, the total cost of hospitalization due to acute myocardial infarction represented an average annual growth of 30.13% since 2004 [2]. Increasing burden of CHD has become a major public health problem; thus, improvements in the prevention and treatment of CHD are urgently needed.

Traditional Chinese medicine (TCM) has always been an essential part of the health care service system in China. It is well known that TCM practitioners usually prescribe herbal formulas or provide other TCM therapies based on Zheng differentiation of every patient using four diagnostic methods, including inspection, listening and smelling, inquiry, and palpation of the pulses. For CHD, a high-quality systematic review [3] concluded that treating CHD with some of the herbs used in TCM might be effective in alleviating angina symptoms, myocardial perfusion abnormalities, or neurological deficit. Furthermore, TCM might have the potential benefit of reducing major adverse cardiac events [4]. In addition, a large number of studies pertaining to the mechanism of herbal medications have shown that the herbs used to treat CHD address such functions as dilating blood vessels, improving microcirculation, reducing blood viscosity, regulating blood lipids, and enhancing energy metabolism and anti-inflammation [5].

However, it is worth mentioning that if TCM doctors had a comprehensive understanding of the difference in Zheng distribution of different types of CHD and stratifications of the Global Registry of Acute Coronary Events (GRACE) score, there might be less therapeutic error, and treatments might be more target-specific. Based on the degree of stenosis and treatment options, coronary stenosis can be divided into 5 types, including atherosclerosis with less than 30% stenosis, borderline coronary lesion with 30% to 70% stenosis, scheduled to undergo percutaneous coronary intervention (PCI), scheduled to undergo coronary artery bypass grafting (CABG), and being unable to undergo revascularization. There are different types of CHD, such as borderline artery lesion, acute coronary syndrome needing PCI and severe artery stenosis demanding CABG, and different stratifications of GRACE score, and these varying types mean different pathology, prognosis, and clinical risk. Thus, obtaining the association between TCM Zheng and types of coronary artery stenosis and GRACE score is crucial to advance the treatment of CHD by TCM. Although some of the recent studies focused on Zheng distributions in CHD patients based on investigations using clinical epidemiology, the association between TCM Zheng and types of coronary artery stenosis and GRACE score remains obscure. Therefore, we planned to carry out a multicenter large-sample case series study among four tertiary A hospitals in China to detect the association between TCM Zheng and types of coronary artery stenosis and GRACE score.

## 2. Objective

The study aims to determine the association between TCM Zheng and types of coronary artery stenosis and to determine the association between TCM Zheng and GRACE score.

## 3. Methods and Design

### 3.1. Study Design

**Figure 1:**
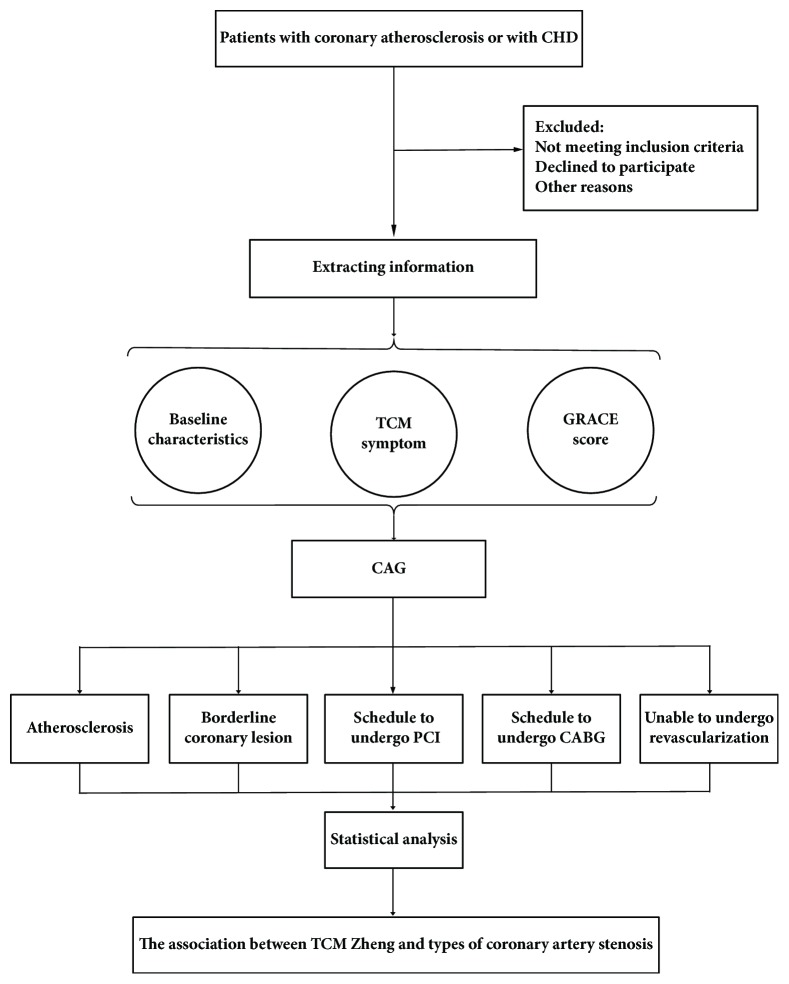
Study flow chart. CHD: coronary heart disease; TCM: traditional Chinese medicine; CAG: coronary angiography; PCI: percutaneous coronary intervention; CABG: coronary artery bypass grafting.

This study is a multicenter, large sample, case series study. The Declaration of Helsinki and Good Clinical Practice (GCP) Guidelines are regarded as the principle of this clinical study. The protocol, informed consent, and electronic case report form (eCRF) of this study have been reviewed and approved by the Ethics Committee of Guang'anmen Hospital of the China Academy of Chinese Medical Sciences (no. 2017-058-KY-01). The participants' information and privacy will be strictly protected. Written informed consent will be obtained from each participant before the clinical information is collected. This study was registered at the Chinese Clinical Trial Registry on November 2, 2017, in both Chinese and English editions; the registration number was ChiCTR-ROC-17013221. The study design is briefly described in Figure 1.

### 3.2. Subject Recruitment

The patients had a diagnosis of coronary atherosclerosis or CHD. Normally, CHD includes borderline coronary lesion, stable angina pectoris (SAP), unstable angina (UA), non-ST-segment-elevation myocardial infraction (NSTEMI), and ST-segment-elevation myocardial infraction (STEMI); these conditions will be included in this case series study. All the patients will be enrolled from November 2017 to December 2018 at four hospitals, identified in Table 1. Three thousand eligible participants will be recruited from these four sites according to the inclusion and exclusion criteria.

### 3.3. Diagnostic Criteria

#### 3.3.1. Traditional Chinese Medicine Diagnostic Criteria of CHD Zheng

TCM Zheng of each participant will be estimated by two TCM experts referring to the “National Standard Clinical Terminology of Traditional Chinese Medical Diagnosis and Treatment–Syndromes (GB/T 16751.2-1997)” and “Traditional Chinese Medicine Syndrome Differentiation Criteria of Coronary Heart Disease” [6]. These two diagnostic criteria involve all the simple Zheng. Permutations and combinations of these simple Zheng are flexible and could encompass all the complex clinical settings. The interrater reliability of diagnosing TCM Zheng will be evaluated using the kappa statistic.

#### 3.3.2. Diagnostic Criteria of CHD

The diagnostic criteria of different types of CHD will refer to the “Guideline for the Diagnosis and Management of Patients with Stable Ischemic Heart Disease” [7] (2012 edition), the “Guideline for the Management of Patients with Non-ST-Elevation Acute Coronary Syndromes” [8] (2014 edition), and the “Guideline for the Management of ST-Elevation Myocardial Infarction” [9] (2013 edition). To establish whether patients with CHD undergo revascularization (PCI or CABG) and whether patients are unable to undergo revascularization due to contraindications, we will refer to “Guidelines on Myocardial Revascularization” [10] (2011 edition). Specifically, we list the indications for revascularization of stable coronary artery disease (SCAD) patients and recommendations for the type of revascularization of SCAD patients in Tables 2 and 3, respectively.

### 3.4. Inclusion and Exclusion Criteria

#### 3.4.1. Inclusion Criteria

Inclusion criteria include the following:

1. Patients with coronary atherosclerosis or with CHD.
2. Planned coronary angiography (CAG).
3. Participant aged from 18 to 80 years.
4. Participants who voluntarily signed the informed consent.

#### 3.4.2. Exclusion Criteria

Exclusion criteria include the following:

1. Participants with acute infection, trauma, burns, and surgical history in the previous week.
2. Participants who have already received PCI or CABG or have received other vascular (including cardiocerebral vascular and peripheral vascular) interventional treatments.
3. Uncontrolled hypertension (occasional resting blood pressure higher than 160/100 mmHg in one week, under drug control), severe ventricular arrhythmia, and III-degree atrioventricular block.
4. Other heart diseases such as rheumatic heart disease, pulmonary heart disease, hyperthyroidism heart disease, dilated cardiomyopathy, hypertrophic cardiomyopathy, severe valvular disease, myocarditis, and pericarditis.
5. Participants with aortic dissection, aortic aneurysms, pulmonary embolism, and severe myocardial bridge.
6. Participants with acute exacerbation of chronic obstructive pulmonary disease and respiratory failure.
7. Participants with liver dysfunction and alanine aminotransferase (ALT)>3 times the normal value or combined with cirrhosis; with kidney dysfunction and estimated glomerular filtration rate (eGFR)<30 mL/min/1.73 m^2^.
8. Participants with hematopoietic system disease, malignant tumor, autoimmune disorders, and infectious disease (tuberculosis, viral hepatitis, syphilis, and acquired immune deficiency syndrome).
9. Participants with acute cerebral stroke and severe complications of diabetes (diabetic foot, diabetic ketoacidosis).
10. Participants with organ transplantation.
11. Participants with psychosis or dysgnosia.
12. Pregnant or lactating women.

### 3.5. Sample Size

In the multifactor statistical analysis, the sample size is usually 5-10 times of the number of variables [11]. In actual use, there have been some false-positive and false-negative errors. In addition, to improve accuracy, the overall sample size is usually designed to be 20-30 times of the number of variables (items in the scale). This study contains approximately 100 variables. Considering a nonresponse rate of at least 15%, the final overall sample design is estimated as 100 × 25 × (1 + 15%) ≈ 3000 cases, with a 95% confidence interval and 2% relative error.

### 3.6. Observational Content

The observational content would include (1) demographics: age, sex, home address, education, height, and weight; (2) life style: smoking history and alcohol consumption history; (3) past medical history; (4) medication history; (5) TCM symptoms: the main complaint, feeling of coldness or fever, perspiration, discomfort in parts of body from head to toe, appetite, quality of sleep, stool, urine, the condition of the tongue and pulse, and so on; (6) TCM Zheng; (7) clinical diagnosis; (8) CAG result; and (9) GRACE score.

## 4. Quality Control

Quality control is crucial in a multicenter study to ensure the accuracy and authenticity. Quality control measures chiefly include organization structure, investigator training, standard operating procedures (SOP), and supervision and internal inspection.

### 4.1. Organization Structure

The multicenter clinical study will be directed and coordinated by the project leader. In addition, the principle investigator (PI) of every site will be responsible for the assignment of work and the quality control of this study. Meanwhile, a study monitor, who is not directly involved in participant enrollment and information collection, will be authorized by the project leader to supervise this study.

### 4.2. Investigators Training

Before starting the clinical study, all the investigators should participate in strict and systematic training to ensure the accuracy of participant information. We will carry out intensive training or online video training for all investigators; the training contents include (1) aim, method and SOP; (2) the standardized process for completing the clinical questionnaire; (3) eCRF usage; and (4) information and data management.

### 4.3. Standard Operating Procedures

#### 4.3.1. Completing the Clinical Questionnaire

(1) To be familiar with the operating procedures, the investigators are expected to read the entire content of the eCRF, instructions on program implementation, and the investigator's handbook carefully before starting the clinical study. (2) Each investigator will have his own separate eCRF account (website: www.medresman.org) to timely, accurately, and thoroughly record each item according to a unified recording method. (3) After recording is complete, each investigator should double-check the information to ensure that there are no omissions or misinformation. (4) If any content in the questionnaire needs to be modified, investigators should indicate the reason for the revision. In addition, any modification process will be recorded by eCRF.

#### 4.3.2. Collecting TCM Symptoms

(1) In the process of collecting clinical information, the investigator should listen patiently and record information carefully. In addition, language should be easily understood by patients, and the use of complicated terminology should be avoided. (2) Each symptom will be divided into four degrees, including “no”, “light”, “medium”, and “heavy”. The instruction of how to classify the degree of each symptom is included in the investigator's handbook. (3) When observing the body and tongue, the investigator should fully expose the parts of the body and examine them under nature light, avoiding the use of colored light. Each tongue should be observed for no more than 30 seconds. If the primary observation of the tongue is not confirmed, patients should rest for 3-5 minutes and investigator may repeat the observation. At this point, for the tongue diagnosis, investigators may refer to the color map in their handbook. (4) Pulse diagnosis is conducted with the radial artery by an experienced TCM practitioner.

#### 4.3.3. Collecting CAG Result and GRACE Score

The CAG result will be judged by the imaging physician. The unified quality control method will be employed to guarantee the accuracy of the GRACE score of each participant, according to international grading standards [12].

#### 4.3.4. Information Management

(1) Patient code: every site has a 2-digit code (Table 1). According to the patient's enrollment order, the corresponding code will be produced. For instance, when Guang'anmen Hospital recruits the first patient, the code will be 01-0001. (2) Dynamic management: by using eCRF, the registration information of the patient will be uploaded synchronously, which will make it simple for the PI to check it in real time. (3) Data collection: to ensure the objectivity and accuracy of the collected data, we recommend that at least two investigators oversee data collection. The diagnosis of the tongue and pulse must be performed by at least two experienced TCM practitioners, and the CAG results should be interpreted by at least two experienced imaging physicians. If there is any disagreement, it will be resolved by discussion and be arbitrated by a third physician. (4) Data entry: all data must be completed by the investigators using the eCRF procedure within 24 of enrollment to ensure accuracy of the data. (5) Data submission: before submitting the eCRF, it should be approved by the PI in charge of the specific site.

### 4.4. Supervision and Internal Inspection

(1) The monitor will randomly conduct on-site or telephone supervision of the sites and ensure that the study is strictly following the protocol. (2) Content for supervision includes informed consent, standardized collection of clinical information, and accuracy of the raw data. (3) The PI of each site should regularly conduct internal inspections to find and resolve problems in timely manner.

## 5. Statistical Analysis

All data will be exported in an Excel-format through the www.medresman.org. The methods for statistical analysis include frequency analysis, factor analysis, cluster analysis, decision tree, and other relevant analytic methods. SPSS 20.0 will be used for statistical analysis.

## 6. Discussion

**Figure 2:**
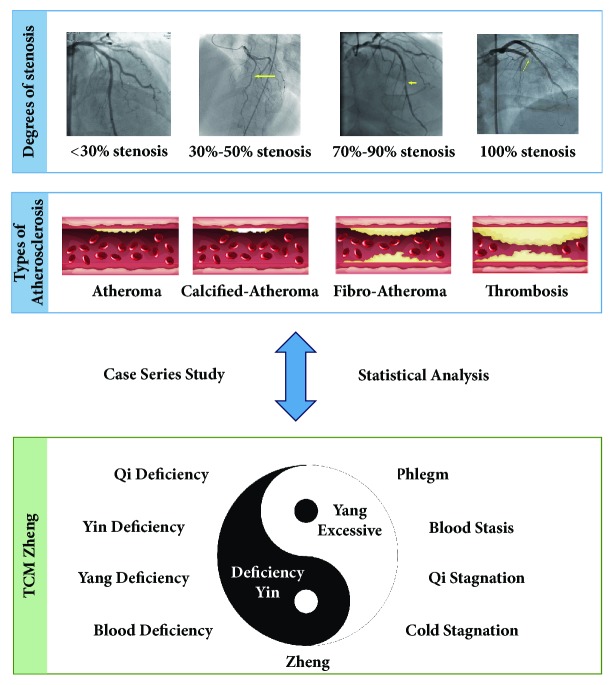
The possible association between TCM Zheng and coronary artery stenosis.

CHD is usually caused by atherosclerosis, which leads to coronary artery stenosis and myocardial ischemia. The treatment strategies for CHD include mainly drugs, PCI, and CABG. It is notable that different degree of coronary artery stenosis may bring about different clinical manifestations and outcomes. In general, patients with atherosclerosis do not have symptoms of chest pain; patients with borderline coronary lesion could manifest as stable angina; patients scheduled to undergo PCI or CABG or those unable to undergo revascularization have similar symptoms including acute coronary syndrome. Studies have indicated that some kinds of TCM Zheng, such as Qi deficiency, Yin deficiency, Yang deficiency, blood stasis, phlegm, and Qi stagnation, may be found in patients with CHD [13]. Within the framework of TCM theory, different TCM Zheng has different clinical manifestations. For instance, the CHD patient with Qi deficiency could present with chest distress, shortness of breath, lassitude, spontaneous sweating, pale tongue with white coating, and weak pulse. In addition, the CHD patient with blood stasis could manifest symptoms such as a steady stabbing pain in the chest, dark complexion, cyanotic lips and nails, scaly skin, subcutaneous ecchymosis, dark and purple tongue with ecchymosis or spots, thread, and rough or knotted and intermittent pulse. Coronary artery stenosis involves several pathophysiological aspects such as endothelial injury, lipid accumulation, intraplaque hemorrhage, platelet aggregation, and thrombogenesis, which, from the perspective of TCM Zheng, could be prevented; blood stasis and phlegm could be addressed within the framework of TCM theory [14]. Previous cross-sectional studies have often analyzed the relationship between TCM Zheng and CHD, and the inclusion criteria usually used computed tomography angiography to diagnose CHD [15]. However, in this study, we focus on CHD patients who are diagnosed by the golden standard of CAG. We are expanding the population of participants to include not only CHD patients with more than 50% coronary stenosis but also patients with simple atherosclerosis and those with 30%-50% coronary stenosis [16]. In recent years, relevant studies have found that some patients with ACS are diagnosed with normal arteries, which may result from microvascular disease and endothelial dysfunction [17]. Therefore, we hope to see if differences among varying degrees of coronary artery stenosis can be identified in TCM Zheng, which may help indicate if TCM Zheng could identify risk factors for the progression of coronary artery stenosis. The possible association between TCM Zheng and coronary artery stenosis is shown in Figure 2. Among the studies assessing TCM Zheng in CHD, we are the first to assess the GRACE score of participants, which may predict long-term cardiovascular endpoint events [18] and identify those associated with different TCM Zheng.

## Figures and Tables

**Table 1:** The hospitals participating in this study.

Code	Participating Center	Code	Participating Center
01	Guang'anmen Hospital, China Academy of Chinese Medical Sciences	03	Yunnan Provincial Hospital of Traditional Chinese Medicine
02	Beijing Anzhen Hospital Capital Medical University	04	The First Affiliated Hospital of Xinxiang Medical University

**Table 2:** Indications for revascularization in patients with stable coronary artery disease.

Extent of CHD	Class	Level
For prognosis		
Left main disease with stenosis >50%	I	A
Any proximal LAD stenosis >50%	I	A
Two-vessel or three-vessel disease with stenosis >50% with impaired LV function (LVEF<40%)	I	A
Large area of ischaemia (>10% LV)	I	A
Single remaining patent coronary artery with stenosis >50%	I	B
For symptoms	I	C
Any coronary stenosis >50% in the presence of limiting angina or angina equivalent, unresponsive to medical therapy	I	A

LAD: left anterior descending coronary artery; LV: left ventricular.

**Table 3:** Recommendation for type of revascularization (CABG or PCI) in patients with stable coronary artery disease.

Extent of CHD	CABG		PCI	
	Class	Level	Class	Level
One- or two-vessel disease without proximal LAD stenosis	IIb	C	I	C
One-vessel disease with proximal LAD stenosis	I	A	I	A
Two-vessel disease with proximal LAD stenosis	I	B	I	C
Left main disease with a SYNTAX score 22	I	B	I	B
Left main disease with a SYNTAX score 23-32	I	B	IIa	B
Left main disease with a SYNTAX score >32	I	B	III	B
Three-vessel disease with a SYNTAX score 22	I	A	I	B
Three-vessel disease with a SYNTAX score 23-32	I	A	III	B
Three-vessel disease with a SYNTAX score >32	I	A	III	B
